# Darker Skin Tone Increases Perceived Discrimination among Male but Not Female Caribbean Black Youth

**DOI:** 10.3390/children4120107

**Published:** 2017-12-12

**Authors:** Shervin Assari, Cleopatra Howard Caldwell

**Affiliations:** 1 Department of Psychiatry, University of Michigan, Ann Arbor, MI 48109, USA; 2 Center for Research on Ethnicity, Culture and Health, School of Public Health, University of Michigan, Ann Arbor, MI 48109, USA; cleoc@umich.edu; 3 Department of Health Behavior and Health Education, School of Public Health, University of Michigan, 2846 SPH I, 1415 Washington Heights, Ann Arbor, MI 48109, USA

**Keywords:** ethnic groups, racism, racial discrimination, race, Blacks, Caribbean Blacks, gender, bias, skin tone

## Abstract

Background: Among most minority groups, males seem to report higher levels of exposure and vulnerability to racial discrimination. Although darker skin tone may increase exposure to racial discrimination, it is yet unknown whether skin tone similarly influences perceived discrimination among male and female Caribbean Black youth. Objective: The current cross-sectional study tests the role of gender on the effects of skin tone on perceived discrimination among Caribbean Black youth. Methods: Data came from the National Survey of American Life-Adolescent Supplement (NSAL-A), 2003–2004, which included 360 Caribbean Black youth (ages 13 to 17). Demographic factors (age and gender), socioeconomic status (SES; family income, income to needs ratio, and subjective SES), skin tone, and perceived everyday discrimination were measured. Linear regressions were used for data analysis. Results: In the pooled sample, darker skin tone was associated with higher levels of perceived discrimination among Caribbean Black youth (*b* = 0.48; 95% Confidence Interval (CI) = 0.07–0.89). A significant interaction was found between gender and skin tone (*b* = 1.17; 95% CI = 0.49–1.86), suggesting a larger effect of skin tone on perceived discrimination for males than females. In stratified models, darker skin tone was associated with more perceived discrimination for males (*b* = 1.20; 95% CI = 0.69–0.72) but not females (*b* = 0.06; 95% CI = −0.42–0.55). Conclusion: Similar to the literature documenting male gender as a vulnerability factor to the effects of racial discrimination, we found that male but not female Caribbean Black youth with darker skin tones perceive more discrimination.

## 1. Introduction

Racial discrimination is one of the main contributors to racial health disparities in the United States [1,2,3,4,5,6,7,8,9]. Across all age groups, Blacks perceive high levels of discrimination [10,11,12,13,14,15], which diminishes their physical and mental health [16,17,18,19]. Perceived discrimination evokes negative emotions such as sadness, anger, and worries [20]. As a result, perceived discrimination is a risk factor for depression [21], anxiety [22], and psychological distress [23]. Discrimination also increases hyper-vigilance [11] which may mediate or moderate the effects of perceived discrimination on psychological distress [20]. Individuals who perceive discrimination are more likely to evaluate their social interactions as harassing [24]. Perceived discrimination also increases the likelihood of engaging in high-risk behaviors such as suicide [25], smoking [26], drinking [27], and drug use [5,28,29]. Discrimination also influences the chances of receiving adequate education, employment, and health care [30,31,32,33].

Gender has been shown to shape both perception of and vulnerability to discrimination. Across several racial and ethnic minority groups, males perceive more discrimination than females [13,29,34,35,36]. Gender may also alter the harmful effects of perceived discrimination [21,37,38,39]. For instance, in a cross-sectional study among Arab Americans, perceived discrimination was a stronger risk factor for psychological distress in males than females [23]. In a longitudinal study among African American adolescents, an increase in perceived discrimination predicted an increase in depressive symptoms among males but not females [21], a finding which was replicated over a longer period of time among emerging adults [22]. These studies suggest that exposure and vulnerability to discrimination may not be merely a function of race but the intersection of race, ethnicity, and gender [23,25,37,39]. Such group differences may be due to the unique life history, values, expectations, attributions, and norms in each intersectional sub-group [40].

Skin color is a major determinant of perceived discrimination and health [41]. Similar results have been reported from the United States [42], Puerto Rico [43], Brazil [41], and India [44], among other countries [41,45]. Monk used US data to study the links between skin tone, discrimination, and health among African-Americans. He found that skin tone is a significant predictor of perceived skin color discrimination from whites as well as Blacks, and, these forms of perceived discrimination are key determinants of health outcomes, such as depression and self-rated mental and physical health. His findings suggested that intra-racial health differences related to skin tone (and discrimination) may even exceed disparities between Blacks and Whites [42]. Using data from Brazil, Perreira and Telles found a gradient effect of skin color on self-rated health, with individuals with darker skin colors reporting poorer health. Their study showed that darker skin color influences self-rated health primarily by increasing exposure to class discrimination and low socio-economic status [41].

Although we know that skin color is a determinant of discrimination, very little is known about gender differences and their role on the effects of skin tone on perceived discrimination among Blacks, particularly Caribbean Black youth. Present literature tends to focus on the effects of skin tone among adults, leaving a gap in the literature for adolescents [43]. In addition, most available research on the effects of skin tone on perceived discrimination is about the overall effects of dark skin tones in racial and ethnic groups, and very little is known about intersectional nuances in these regards [43]. Although overall, individuals with darker skin tones generally report more discrimination [44,45,46,47,48], there is some evidence that suggests that this effect may depend on gender [43]. Thus, there is a need to study the intersection of ethnicity and gender on the effects of skin tone on perceived discrimination among Caribbean Black youth.

The current study tested whether gender alters the effects of skin tone on perceived discrimination using a national sample of Caribbean Black youth. In line with the literature on higher exposure and vulnerability to discrimination among males [49,50], we expected a stronger effect of skin tone on perceived discrimination among male compared to female Caribbean Black youth.

## 2. Methods

### 2.1. Design and Setting

Using a cross-sectional design, data came from the National Survey of American Life-Adolescent Supplement (NSAL-Adolescents) [13,51]. The NSAL-A was conducted as part of the Collaborative Psychiatric Epidemiology Surveys (CPES), 2001–2003 [52]. The NSAL-A is one of the few available national mental health surveys on Black American youth [53].

### 2.2. Ethics

The NSAL study protocol was approved (B03-00004038-R1, approved 2003) by the University of Michigan Institutional Review Board (IRB). Adolescents’ legal guardians signed written informed consent. All adolescents provided assent themselves. All the respondents received financial compensation ($50). Funded by the National Institute of Mental Health (NIMH), the study was conducted by the University of Michigan (UM), Ann Arbor.

### 2.3. Participants

The current analysis included 360 Caribbean Black youth who were recruited in the NSAL-A. We did not include the remaining 810 African American youth who participated in the NSAL-A because our interest was in results for Caribbean Black youth. All of the participants were between the ages of 13 and 17. All participants resided in territories of the United States at the time of the survey. More detailed information on the sampling strategy is available elsewhere [48].

All participants in the current analysis belonged to the Caribbean Black ethnicity. NSAL-Adolescents collected data on the self-assigned ethnicity of the family household in which the adolescent lived. Parents of the households identified their ethnicity as African American or Caribbean Black. Caribbean Black was defined as Blacks having ancestral ties to a country included on a list of Caribbean countries provided by the interviewer or Blacks whose parents or grandparents were born in any of the following Caribbean countries: Antigua and Barbuda, Barbados, Bahamas, Cuba, Dominican Republic, Dominica, Grenada, Haiti, Jamaica, Saint Vincent and the Grenadines, Trinidad and Tobago, Saint Lucia, and Saint Kitts and Nevis.

### 2.4. Sampling

The NSAL-A sample lived in the same households as the NSAL-Adult sample. The NSAL-Adult sample used a national probability sample of African American and Caribbean Black households in the United States. The NSAL-Adults households were screened for eligible adolescents living in the same household. Adolescents who were living in the households were randomly selected for participation in the NSAL-Adolescents. If more than one eligible youth were living in a household, two adolescents were selected based on the gender of the first eligible youth. This strategy resulted in non-independence for adolescent samples. In response to this lack of independence, the adolescent supplement data was weighted to adjust for non-independence in the selection probabilities and non-response at the household and individual levels. The weighted data were then post-stratified to represent national estimates that were based on age, ethnicity, and gender [17].

### 2.5. Interviews

All interviews were conducted in English. Interviews lasted for 100 min on average. The overall response rate for the NSAL-A was 80.6%. The response rate was 83.5% for Caribbean Black youth, which is 3.1% higher than the response rate for the African American youth.

In about 82% of the interviews, in-person interviews were used for data collection. These interviews were performed in the adolescents’ homes. The remaining 18% of the interviews were conducted either entirely or partially by telephone.

Computer-assisted personal interviews (CAPIs) were used for in-person interviews. In CAPI, computers were used by trained interviewers to conduct interviews. CAPI is one of the preferred interviewing methods for complex questionnaires that are time consuming [54].

### 2.6. Measures

*Demographic Characteristics*. The study included demographic factors including age and gender. Gender was self-reported and treated as a dichotomous factor with male as the reference category. Age was treated as a continuous measure.

*Socioeconomic Status (SES)*. SES indicators including family income, income to needs ratio, and subjective SES were measured. Income to needs ratio was defined based on the 2001 US Census, which divides household income by the poverty threshold [55]. Higher scores of all measures were indicative of higher SES.

*Perceived Discrimination*. NSAL-A used a 13-item version of the Everyday Discrimination Scale (EDS) to measure perceived discrimination. This measure is designed to assess chronic, routine, and less overt discriminatory experiences that occurred during the past year [56]. Although the original EDS only includes 10 items, the NSAL-A included three additional items to cover perceived teacher discrimination as well. Though the EDS was originally developed and normed among adults, it has shown good psychometric properties for adolescents as well [17,43,57]. Some of the sample items are: “being followed around in stores”, “people acting as if they think you are dishonest”, “receiving poorer service than other people at restaurants”, and “being called names or insulted”. The responses used a Likert scale ranging from 1 (never) to 6 (almost every day). A sum score was calculated that reflected frequency of exposure to discrimination over the past year. (α = 0.86)

*Skin Tone*. Skin tone (skin complexion) was measured using a single item self-reported measure. Participants were asked to evaluate their skin tone using five categories: 0 (“very light brown”), 1 (“light brown”), 2 (“medium”), 3 (“dark brown”), and 4 (“very dark brown”). This measure strongly correlates with interviewers’ evaluations of skin tone (correlation coefficient = 0.80), suggesting that self-report is a valid measure. Higher scores were indicative of darker skin tones [43].

### 2.7. Statistical Analysis

To accommodate the complex design of NSAL-A, we used Stata 13.0 (Stata Corp., College Station, TX, USA) for data analysis. We used the Taylor expansion approximation technique to re-calculate the complex design-based estimates of variance. Standard errors reflect the weights due to the complex sampling design. We reported frequencies, means, as well as Pearson correlation r values. Unstandardized regression coefficients (*b*) and their 95% Confidence Intervals (CI) were reported for multivariable analysis. *p* values smaller than 0.05 were considered as statistically significant. Missing data were not imputed. Complete case analysis was used for regression models.

Sub-population survey linear regressions were used for our multivariable analyses. In the linear regression models, perceived discrimination was the dependent variable, skin tone was the independent variable, and age, gender, and SES indicators (family income, income to needs ratio, and subjective SES) were covariates. In the first step, the association of interest was estimated in the pooled sample of Caribbean Black youth (Model 1). In the next step, an interaction term between skin tone and gender was added to the model (Model 2). Finally, models specific to each gender were run (Model 3 and Model 4).

## 3. Results

### 3.1. Descriptive Statistics

Table 1 summarizes the descriptive statistics of the participating Caribbean Black youth. Perceived discrimination was higher in males than females. Although Caribbean Black males reported themselves as being slightly darker than Caribbean Black females, the difference was not significant.

### 3.2. Bivariate Associations

Table 2 shows the results of bivariate correlations between gender, age, SES indicators, skin tone, and perceived discrimination in the pooled sample. Skin tone and perceived discrimination were positively correlated in the pooled sample (Table 1).

Table 2 also shows the results of bivariate correlations between age, SES indicators, skin tone, and perceived discrimination separately for male and female Caribbean Black youth. Skin tone and perceived discrimination were positively correlated in male but not female Caribbean Black youth (Table 2).

### 3.3. Linear Regression Models in Caribbean Black Sample

Table 3 summarizes the results of two linear regressions in the pooled sample of Caribbean Black youth, with skin tone as the independent variable, perceived discrimination as the dependent variable, and age, gender, and SES indicators as covariates. Model 1 only included the main effects. Model 2 also included an interaction term between gender and skin tone.

In the pooled sample of Caribbean Black youth, darker skin color was associated with more perceived discrimination (*b* = 0.48; 95% Confidence Interval (CI) = 0.07–0.89). A significant interaction was found between the effects of gender and skin tone on perceived discrimination (*b* = 0.1.17; 95% CI = 0.49–1.86), suggesting that skin tone has a larger effect on perceived discrimination among Caribbean Black males than Caribbean Black females (Table 3).

### 3.4. Linear Regression Models Based on Gender

Table 4 presents the results of Model 3 and Model 4 with skin tone as the independent variable and perceived discrimination as the dependent variable in each gender. In this stratified sample, darker skin color was associated with more perceived discrimination in Caribbean Black males (*b* = 1.20; 95% CI = 0.69–0.72) but not in Caribbean Black females (*b* = 0.06; 95% CI = −0.42–0.55) (Table 4).

## 4. Discussion

This study presented two new findings. First, a darker skin tone is associated with more perceived discrimination among Caribbean Black youth. Second, this effect is present for male but not female Caribbean Black youth.

While research has consistently shown that ethno-racial minorities perceive more discrimination than whites, far less is known about intra-racial heterogeneity in perceived discrimination. In this study, taking an intersectionality approach, we focused on a neglected area in research on skin tone bias against Caribbean adolescent youth. Our finding that Black males are more vulnerable to the effects of skin tone on experiences of discrimination than Black females may be due to racial profiling and threat-based discrimination of Black males. We do not attribute our findings to ethnicity as all of our participants were Caribbean Blacks, and ethnicity did not vary in our study.

This study highlights the role of gender and ethnicity as a central construct for studying the effects of race on health [12]. Based on this study, discrimination is most commonly perceived by male Black youth with the darkest skin tones. As discrimination is inhumane and immoral [58], and as it has a wide range of negative health consequences [1,2,3,4,5,6,7,8,9,10,11,12,13,14,15,16,17,18,19]. There is a need for policies and programs that reduce discrimination for all groups of minorities, particularly Black males who are at the highest risk of both exposure and vulnerability to discrimination. Examples of policies and programs include reducing discrimination against Black males at schools and shops [59]. Black men and boys are disproportionately discriminated against by the educational system, correctional system, police, and labor market [59].

These findings add to the existing literature on gender differences in exposure and vulnerability to race related stress and discrimination. Among African Americans, perceived discrimination and environmental stressors have larger effects on depression and substance use among males than females [60]. In a recent study among Caribbean Black youth, males were more vulnerable than females to the effects of perceived discrimination on substance use [61]. Other research has also shown that discrimination better predicts substance use in Black males than Black females [39]. In another study, recent experience of discrimination increased the risk of smoking for male but not female African Americans [62]. Similar gender differences in the effects of discrimination are reported for other domains of psychopathology such as psychological distress [23], depressive and anxiety symptoms [21,22], and major depressive disorder (MDD) [60]. These studies collectively suggest that males are the main victim of racial discrimination, and they are more vulnerable to it. That means, racial discrimination may be a more salient risk factor for psychopathology of male than female minorities.

Our findings may be explained by how media portrays Black males, and how media shapes stereotypes at a societal level. US media has historically portrayed Black males as aggressive and anti-intellectual [35,36,61,62]. As a result, Black men have been stereotyped as “endangered, aggressive, angry, superhuman, subhuman, lazy, hyperactive, jailed, and paroled, on probation, lost, loveless, incorrigible, or just simply self-destructive” [63,64]. These stereotypes evoke even more discrimination when a Black male has a larger body size or a darker skin tone.

Our findings introduce skin tone as a triggering factor for discrimination against Caribbean Black males. This discrimination may include discrimination carried out by the correctional system and the police. Black males are disproportionately affected by police brutality, mass incarceration, and stop and frisk [65,66,67,68]. Police may introduce new trainings to its system that reduce blunt reactions toward Black males, particularly toward those with larger body sizes and darker skin tones. The same applies to school teachers and principals, as they may also discriminate more against male Black youth with darker skin tones [69,70,71]. Discrimination at school is one of the mechanisms that explains why Black males have a high rate of school drop-out and why they are at risk of school to prison pipeline [72,73,74,75].

The findings of this study suggest that parents should ensure that their race socialization messages should reflect not only gender but also the skin tone of their children. Research has shown that parents already provide more race socialization messages for Black boys than Black girls [76,77]. The results of this study would argue that these messages should extend to include the skin tone (and possibly body size) of Black youth.

Ultimately, it is not only gender differences that exist in exposure and vulnerability to discrimination, with Black males receiving the highest level of exposure [37] and being most vulnerable to discrimination [21]; gender also determines whether dark skin tone increases the level of perceived discrimination or not. Although female Caribbean Black youth experience discrimination regardless of their skin tone, that is not the case for Caribbean Black males.

Theoretical frameworks provide a coherent explanation for gender differences in exposure and vulnerability to discrimination. The subordinate male target hypothesis argues that Black men are subject to more experiences of discrimination [49]. According to this model, social patterning of discrimination does not only depend on race or ethnicity but the intersection of race, ethnicity, and gender. Appending the model, our results suggest that discrimination is shaped by the intersection of race, ethnicity, gender, and skin tone (and probably facial characteristics, and body size).

We still do not know whether skin tone reflects actual encounters of discrimination, or it increases attribution of ambiguous exposures to discrimination. Skin tone may be related to cumulative exposure to discrimination, vigilance, racial identity, and social class which all shape norms and values about race and gender. A considerable amount of literature suggests that class [11,78,79,80,81], racial identity [5,15,36,38,82,83], and gender norms [36,40] influence exposure and vulnerability to discrimination. Attribution of ambiguous situations to discrimination depend on the mental model, racial identity, and salience of race in daily encounters [29,82]. Masculine ideologies and gender norms may also explain why some of these effects are stronger for males than females. Stress and discrimination are shown to have stronger negative effects when hegemonic masculinity is high [57,83]. Expectations about dominance and hierarchy may change how people experience and respond to discrimination [36,40]. Strong masculine beliefs make individuals vulnerable to discrimination and related social stress [57,83]. Gender also influences coping strategies [36,84]. Overall, men have a higher tendency to use confrontational coping [85] compared to women who have a higher tendency to use avoidant coping [84]. In contrast to women, men have a higher tendency to act out their stress [86] and to externalize their emotions [87]. These findings may explain why gender alters perceived discrimination among minorities. Among Blacks, high SES may be associated with an increased vulnerability to discrimination [79,88]. For example, Hudson et al., showed that high SES may increase Black men’s vulnerability to perceived discrimination [88]. Another study also showed that high subjective SES increases the vulnerability to perceived discrimination [79]. These findings may explain the positive association between SES and psychological distress in Black men [89,90,91,92,93,94]. These findings may collectively why SES generates less health for Blacks, compared to Whites, a phenomenon also called as Blacks’ diminished return [95,96].

## 5. Limitations

The current study had a few limitations. First, data were collected about 15 years ago. Still, NSAL-A is one of the most recent data sets that has data on Caribbean Black youth. In this study, we did not include measures of attribution or discrimination due to gender, and other factors. We also did not include immigration data. Future research should test whether these effects are similar for first and second-generation Blacks or not. Additional research that includes measures of generation may highlight the assimilation or selection effects. Despite these limitations, having a national sample, large sample size of Caribbean Black youth, and taking an intersectional approach were among the strengths of the study.

## 6. Conclusions

Similar to the literature that suggests that gender alters exposure and vulnerability to discrimination, this study documented gender differences in the effects of skin tone on perceived discrimination among Caribbean Black youth. The result shows that Caribbean Black males with darker skin tones perceive more discrimination compared to their lighter skin counterparts (and Caribbean Black females). Although there is a need to reduce racism in the United States at all levels, discrimination may have a larger role for psychopathology of Black males than Black females.

## Figures and Tables

**Table 1:** Summary of descriptive statistics in the pooled sample of Caribbean Black youth and based on gender.

Characteristics	Caribbean Blacks All		Caribbean Black Males		Caribbean Black Females	
	Mean	95% CI	Mean	95% CI	Mean	95% CI ^b^
Age (Year) ^a^	15.21	15.08–15.34	14.80	14.59–15.01	15.55	15.44–15.65
Income ($1000) ^a^	0.58	−8.08–9.25	1.77	−7.23–10.77	−0.40	−8.92–8.11
Subjective Socioeconomic Status ^a^	2.17	2.12–2.22	2.26	2.11–2.42	2.09	2.00–2.17
Income to Needs Ratio ^a^	4.19	3.61–4.77	4.43	3.58–5.27	4.00	3.62–4.39
Skin Tone (Darker)	2.07	1.95–2.19	2.09	1.81–2.37	2.04	1.61–2.48
Perceived Everyday Discrimination ^a^	5.22	4.03–6.41	6.13	4.25–8.01	4.48	3.75–5.22

^a^ *p* < 0.05; ^b^ CI—Confidence Interval.

**Table 2:** Summary of correlation matrix in the pooled sample of Caribbean Black youth.

Characteristics		1	2	3	4	5	6	7
**No.**	**All Caribbean Blacks**							
1	Gender (Male)	1.00						
2	Age (Years)	−0.10	1.00					
3	Family Income ($1000)	−0.06	0.00	1.00				
4	Subjective Socioeconomic Status	0.01	0.04	−0.31 *	1.00			
5	Income to Needs Ratio	−0.01	0.03	0.79 *	−0.21	1.00		
6	Skin Tone (Darker)	0.12	0.03	−0.04	0.05	0.02	1.00	
7	Perceived Everyday Discrimination	0.09	0.13	0.06	0.03	0.14	0.17 *	1.00
**No.**	**Caribbean Black Females**							
1	Gender (Male)	-						
2	Age (Years)	-	1.00					
3	Family Income ($1000)	-	−0.02	1.00				
4	Subjective Socioeconomic Status	-	0.08	−0.37	1.00			
5	Income to Needs Ratio	-	−0.04	0.78 *	−0.32 *	1.00		
6	Skin Tone (Darker)	-	0.06	−0.02	0.07	0.05	1.00	
7	Perceived Everyday Discrimination	-	0.10	0.13	−0.03	0.22 *	0.11	1.00
**No.**	**Caribbean Black Males**							
1	Gender (Male)	-						
2	Age (Years)	-	1.00					
3	Family Income ($1000)	-	0.00	1.00				
4	Subjective Socioeconomic Status	-	−0.01	−0.24 *	1.00			
5	Income to Needs Ratio	-	0.10	0.81 *	-0.07	1.00		
6	Skin Tone (Darker)	-	0.03	−0.07	0.02	−0.02	1.00	
7	Perceived Everyday Discrimination	-	0.18	−0.01	0.09	0.05	0.23 *	1.00

* *p* < 0.05.

**Table 3:** Linear regressions in the pooled sample of Caribbean Black youth.

Characteristics	All Caribbean Black Youth			
	Model 1 Main Effects		Model 2 Main Effects and Interaction	
	*b*	95% CI	*b*	95% CI
Gender (Male)	1.36 **	0.52–2.20	−1.08	−2.75–0.60
Age (Years)	0.12	−0.21–0.44	0.13	−0.14–0.40
Family Income ($1000)	−0.02	−0.07–0.03	−0.02	−0.06–0.03
Subjective Socioeconomic Status	0.75 *	0.01–1.48	0.80 **	0.26–1.34
Income to Needs Ratio	0.57	−0.29–1.42	0.59	−0.16–1.35
Skin Tone (Darker)	0.48 *	0.07–0.89	0.06	−0.34–0.47
Skin Tone (Darker) × Gender (Male)	-	-	1.17 **	0.49–1.86
Intercept	−2.11	−4.53–0.32	−1.69	−4.11–0.73

Outcome—Perceived everyday discrimination; *b*—unstandardized regression coefficient; * *p* < 0.05; ** *p* < 0.01.

**Table 4:** Linear regressions in male and female Caribbean Black youth.

Characteristics	Caribbean Black Females		Caribbean Black Males	
	*b*	95% CI	*b*	95% CI
Age	0.25	−0.17–0.67	−0.06	−0.41–0.30
Income	−0.01	−0.05–0.02	−0.03	−0.12–0.05
Subjective Socioeconomic Status	0.43	−0.76–1.62	1.07	−0.30–2.44
Income to Needs Ratio	0.56 *	−0.05–1.18	0.67	−0.46–1.80
Skin Tone (Darker)	0.06	−0.42–0.55	1.20 **	0.69–1.72
Intercept	−2.71	−9.46–4.03	−0.86	−6.84–5.12

Outcome—Perceived everyday discrimination; *b*—unstandardized regression coefficient; * *p* < 0.1; ** *p* < 0.05.
